# Autophagy Protects Advanced Glycation End Product-Induced Apoptosis and Expression of MMP-3 and MMP-13 in Rat Chondrocytes

**DOI:** 10.1155/2017/6341919

**Published:** 2017-02-07

**Authors:** Wenzhou Huang, Peng Ao, Jian Li, Tianlong Wu, Libiao Xu, Zhongbo Deng, Wenjie Chen, Changchang Yin, Xigao Cheng

**Affiliations:** ^1^Department of Orthopaedic Surgery, The Second Affiliated Hospital of Nanchang University, Nanchang, Jiangxi 330006, China; ^2^Jiujiang University, Key Laboratory of Medical Transformation of Jiujiang, Jiujiang, Jiangxi 332000, China

## Abstract

Aging is one of the most prominent risk factors for the pathological progression of osteoarthritis (OA). One feature of age-related changes in OA is advanced glycation end products (AGEs) accumulation in articular cartilage. Autophagy plays a cellular housekeeping role by removing dysfunctional cellular organelles and proteins. However, the relationship between autophagy and AGE-associated OA is unknown. The aim of this study is to determine whether autophagy participates in the pathology of AGE-treated chondrocytes and to investigate the exact role of autophagy in AGE-induced cell apoptosis and expression of matrix metalloproteinase- (MMP-) 3 and MMP-13. AGEs induced notable apoptosis that was detected by Annexin V/PI double-staining, and the upregulation of MMP-3 and MMP-13 was confirmed by Western blotting. Autophagy-related proteins were also determined by Western blotting, and chondrocytes were transfected with mCherry-GFP-LC3B-adenovirus to monitor autophagic flux. As a result, autophagy significantly increased in chondrocytes and peaked at 6 h. Furthermore, rapamycin (RA) attenuated AGE-induced apoptosis and expression of MMP-3 and MMP-13 by autophagy activation. In contrast, pretreatment with autophagy inhibitor 3-methyladenine (3-MA) enhanced the abovementioned effects of AGEs. We therefore demonstrated that autophagy is linked with AGE-related pathology in rat chondrocytes and plays a protective role in AGE-induced apoptosis and expression of MMP-3 and MMP-13.

## 1. Introduction

Osteoarthritis (OA), the most common skeletal disorder, causes chronic pain during joint movement and disability in the elderly [1, 2]. The pathogenesis of OA is very complicated and comprises multiple risk factors, including cell biological, mechanical, genetic, and age-related factors, with aging being the main risk factor [2, 3]. Advanced glycation end products (AGEs) resulting from a nonenzymatic reaction between sugars and proteins are associated with age-related changes in many tissues [4]. The only way to remove AGEs is largely determined by the rate of protein degradation [5]. Articular cartilage is particularly sensitive to the accumulation of AGEs due to the long half-life of type II collagen [6]. In joint tissue, the excessive level of AGEs plays an important role in the development and progression of OA via collagen crosslinking, chondrocyte apoptosis, increased inflammatory cytokines, stimulated expression of matrix metalloproteinases (MMPs), and loss of collagen II [6–8]. The articular cartilage is composed of an extracellular matrix (ECM) and one cell type, chondrocytes. Because apoptosis and catabolism of chondrocytes in the ECM both play pivotal roles in the progress of OA, inhibiting apoptosis and catabolic processes in chondrocytes may prevent the degeneration of articular cartilage.

Autophagy is closely associated with the retardation of aging and therapy for age-related diseases due to its cellular housekeeping role by removing dysfunctional cellular organelles and proteins [9]. However, autophagy declines with aging and during OA. Furthermore, the reduction of autophagy is often accompanied with increases in apoptosis and cartilage degeneration [10]. It has been proposed that autophagy maintains its declination tendency after its peak at the early stage of OA and further causes the aggravation of OA [11]. Thus, augmentation of autophagy may have protective effects on chondrocytes and postpone cartilage degenerative processes in OA. Recent reports show that enhancement of autophagy significantly reduces cartilage degradation by reducing the death of chondrocytes and catabolic factors such as MMP-13 expression [12, 13]. Furthermore, autophagy has been demonstrated to be capable of protecting cardiomyocytes and osteoblasts from AGE-induced apoptosis [14, 15]. However, the role of autophagy in the pathology of AGEs in chondrocytes has not been elucidated. We therefore sought to explore the regulating effect of autophagy on AGE-induced apoptosis and MMP in chondrocytes.

## 2. Materials and Methods

### 2.1. Reagents and Antibodies

Fetal bovine serum (FBS) and Dulbecco's modified Eagle's medium/F12 (DMEM/F12) were purchased from Gibco (Grand Island, NY, USA). AGE-bovine serum albumin (BSA) was purchased from Merck Millipore (Darmstadt, Germany). BSA and the inhibitor of autophagy 3-methyladenine (3-MA) were purchased from Sigma-Aldrich (St. Louis, MO, USA). The inducer of autophagy rapamycin (RA) was purchased from Selleck Chemicals (Houston, TX, USA). Cell Counting Kit-8 (CCK-8) was obtained from Zoman Biotech. Co., Ltd. (Beijing, China). The Annexin V-FITC/PI apoptosis detection kit was purchased from Multi Sciences Biotech. Co., Ltd. (Hangzhou, China). The mCherry-GFP-LC3b-adenovirus was purchased from Beyotime Institute of Biotechnology (Shanghai, China). The GAPDH antibody was obtained from Abcam (Cambridge, MA, USA). Light chain (LC) 3B, Beclin1, MMP-3, and MMP-13 antibodies were purchased from Novus Biologicals (Littleton, CO, USA).

### 2.2. Cell Culture

Sprague-Dawley rats that were 3-4 weeks old were sacrificed by anesthetic overdose. Articular cartilage was isolated from femoral and knee joints of Sprague-Dawley rats under aseptic conditions. The isolated cartilage was dissected into small sections, followed by digestion in 0.25% trypsin at 37°C for 30 min and 0.2% type II collagenase dissolved in DMEM/F-12 without serum at 37°C for 3 h. After filtration, the cells were cultured in complete DMEM/F12 medium (containing 10% FBS and 1% penicillin-streptomycin) at 37°C in a humidified atmosphere containing 5% CO2. Culture medium was changed every other day. When confluent, chondrocytes were detached using 0.25% trypsin and further subcultured. Second-passage cells were maintained in a monolayer and used for experimentation. All animal procedures were approved by the Institutional Animal Care and Use Committee and Ethics Committee of Jiujiang University, Jiangxi, China.

### 2.3. Cell Viability Assay

Chondrocytes were seeded in 96-well plates (5 × 10^3^ cells/well) and incubated in 100 *μ*L complete DMEM/F12 medium for 24 h. Different concentrations of BSA and AGEs were added to the medium. Each treatment was repeated in three wells. For incubation after 24 h, 10 *μ*L of CCK-8 was added to the culture medium. After incubation at 37°C for 2 h, absorbance of the sample was measured at a wavelength of 450 nm with a microplate reader (Biotek, VT, USA).

### 2.4. Flow Cytometric Analysis

The apoptotic incidence was detected using an Annexin V/fluorescein isothiocyanate (FITC) apoptosis detection kit. Chondrocytes were grown in 6-well plates at 2 × 10^5^ cells/well with complete culture medium. After treatments, the attached and supernatant cells were collected together in 500 *μ*L 1x binding buffer and then incubated with 10 *μ*L Annexin V-FITC solution and 5 *μ*L PI solution for 15 min in the dark. Apoptotic cells, including early apoptotic (Annexin V+/PI−) and late apoptotic/necrotic (Annexin V+/PI+) cells, were counted by a FACScan flow cytometer (ACEA Biosciences, CA, USA). All experiments were performed at least three times for each condition.

### 2.5. mCherry-GFP-LC3-Adenovirus Transduction of Chondrocytes

Chondrocytes cultured in 24-well plates (1 × 10^5^ cells/well) were transfected with mCherry-GFP-LC3-adenovirus at 40 MOI for 24 h. After transfection, cells were incubated with complete culture media again for 24 h and then treated with AGEs for the indicated time. The cells were further washed with PBS and fixed with 4% paraformaldehyde for 15 min. The number of GFP and mCherry dots per cell was counted in three randomly selected fields under a fluorescence microscope (Nikon, Kanagawa, Japan). The number of puncta per cell was calculated by dividing the total number of dots by the number of cells in each field.

### 2.6. Western Blot

Total proteins were extracted from chondrocytes with the Total Protein Extraction Kit (Applygen Tech, Beijing, China) according to the manufacturer's instructions. Protein concentrations were measured by the BCA Protein Assay Kit (Solarbio, Beijing, China). In total, 30 *μ*g of proteins was separated by 10–15% sodium dodecyl sulfate polyacrylamide gel electrophoresis (SDS-PAGE) gels and electrophoretically transferred onto polyvinylidene fluoride (PVDF) membranes (Bio-Rad, Hercules, CA). The membranes were blocked with 5% nonfat milk in Tris-buffered saline (TBS) containing 0.1% Tween-20 (TBST) at room temperature for 1 h. The membranes were then incubated overnight at 4°C in 5% nonfat milk in TBST with the primary antibodies previously mentioned. After washing with TBST three times for 30 min, membranes were incubated with IgG-horseradish peroxidase- (HRP-) labeled secondary antibodies (Proteintech, Wuhan, China) at room temperature for 1 h. The blots were detected with an enhanced ECL reagent (Solarbio, Beijing, China) and exposed to X-ray film. ImageJ software 1.48 (Bethesda, MD, USA) was used for the density analysis of protein bands from X-ray film.

### 2.7. Statistical Analysis

The data are presented as the mean ± standard deviation (SD) of at least three independent experiments. The ANOVA test was used for the statistical analysis among multiple groups, followed by a pairwise comparison using Student's *t*-test. *p* < 0.05 was considered to be statistically significant. Statistical analyses were performed using SPSS statistical software 19.0 (IBM, Armonk, NY, USA).

## 3. Results

### 3.1. AGE-Mediated Cytotoxicity and Upregulation of MMPs in Chondrocytes

**Figure 1:**
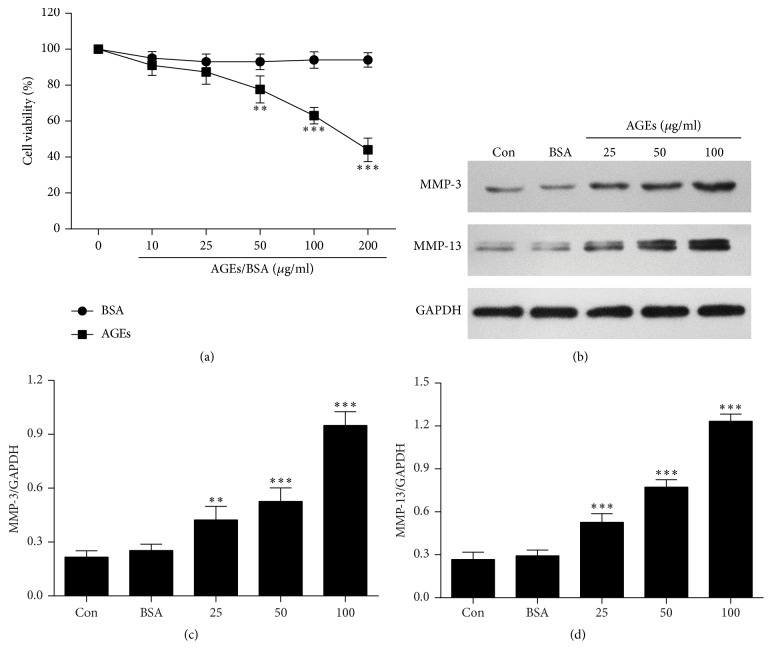
AGEs inhibit cell viability and increase levels of MMP-3 and MMP-13 in chondrocytes. (a) Cell viability assay. Chondrocytes were treated with the indicated dose of AGEs and BSA for 24 h. Cell viability was measured by the CCK-8 assay. (b) Western blot analysis of MMP-3 and MMP-13 after incubating chondrocytes with different concentrations of AGEs for 24 h. (c, d) Protein densitometric quantification of MMP-3 and MMP-13. Dates are presented as the mean ± SD of at least three independent experiments. ^*∗∗*^*p* < 0.01 and ^*∗∗∗*^*p* < 0.001 versus the control group.

The effect of AGEs on cell survival in chondrocytes was evaluated. The CCK-8 assay showed that cell viability decreased in a dose-dependent manner after 24 h treatment with AGEs. At the AGE concentration of 100 *μ*g/mL, a 37% decrease in cell viability was observed. Furthermore, BSA had no significant cytotoxic effect on chondrocytes (Figure 1(a)).

Chondrocytes were treated with AGEs at different concentrations (0, 25, 50, and 100 *μ*g/mL) for 24 h. Western blot analysis showed that AGEs significantly increased protein levels of MMP-3 and MMP-13 in a dose-dependent manner (Figures 1(b), 1(c), and 1(d)). Thus, we choose 100 *μ*g/mL as the optimal concentration of AGEs in subsequent experiments.

### 3.2. AGE-Induced Autophagy in Chondrocytes

**Figure 2:**
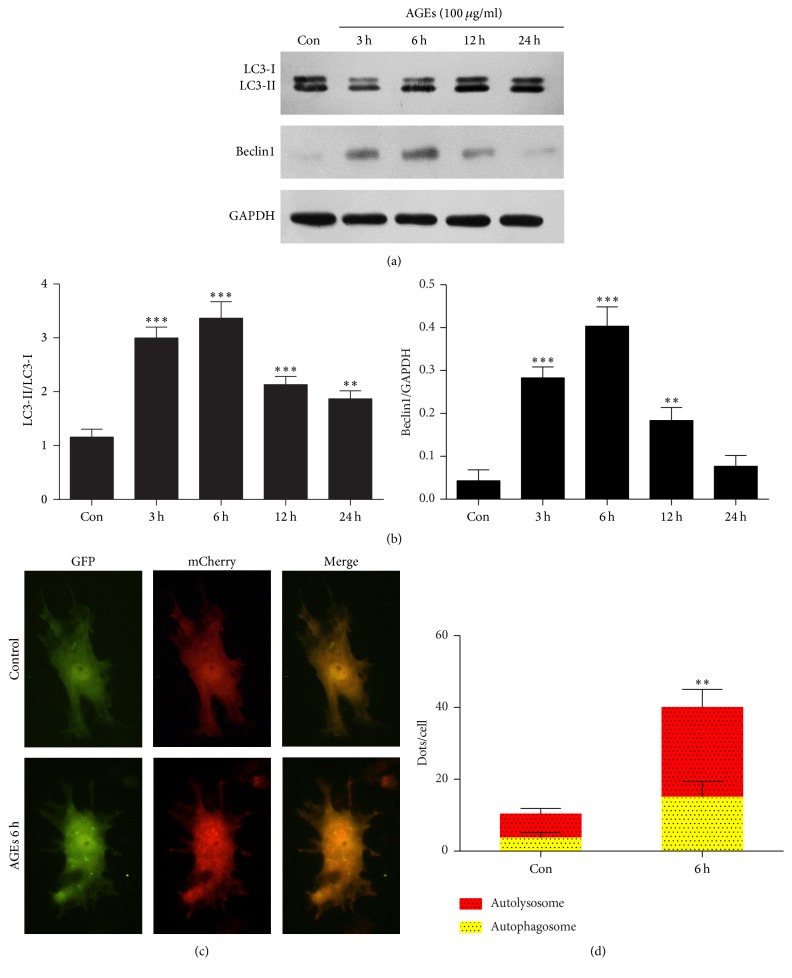
AGEs increase chondrocytes autophagy. (a) Western blot analysis of Beclin1, LC3-I, and LC3-II after incubating chondrocytes with 100 *μ*g/mL AGEs at different time points. (b) Protein densitometric quantification of Beclin1 and LC3-II/LC3-I ratio. (c) Fluorescent microscopy analysis of chondrocytes transfected with mCherry-GFP-LC3, followed by 100 *μ*g/mL AGEs for 6 h. (d) Statistical analysis of fluorescent dots in chondrocytes. Data represent the mean ± SD of three independent experiments. ^*∗∗*^*p* < 0.01 and ^*∗∗∗*^*p* < 0.001 versus the control group.

To determine whether AGEs induced autophagy, we treated chondrocytes with AGEs at various time points. Beclin1 is an essential regulator of the autophagosome formation. Stimulation of Beclin1 triggers nucleation of the autophagic vesicle [16]. LC3 is present in two subtypes, LC3-I and LC3-II. The conversion of LC3-I to LC3-II results in the conjugation between LC3-II and autophagy vesicles, which is associated with autophagosome formation [16, 17]. Thus, the relative expression ratios of LC3-II/LC3-I and Beclin1/GAPDH were used to evaluate the extent of autophagy. Western blot analysis showed that the expression of Beclin1 and the ratio of LC3-II to LC3-I were significantly increased in chondrocytes treated with AGEs, peaking at 6 h and then decreasing gradually (Figures 2(a) and 2(b)).

To ascertain whether the increase of LC3B-II levels was due to activation of autophagic flux or blockade of autophagosome-lysosome fusion, chondrocytes were transfected with adenovirus expressing mCherry-GFP-LC3B to monitor autophagic flux. The GFP signal is unstable in the acidic conditions of the lysosome lumen, whereas mCherry is acid-stable. Thus, colocalization of both GFP and mCherry fluorescence (yellow puncta) indicates that an autophagosome has not fused with a lysosome, whereas a mCherry signal without GFP (red puncta) indicates an autolysosome formation [18]. Fluorescent microscopy showed that AGE treatment increased the number of red puncta, indicating autolysosome formation in chondrocytes (Figures 2(c) and 2(d)).

### 3.3. Autophagy Protects AGE-Induced Apoptosis in Chondrocytes

**Figure 3:**
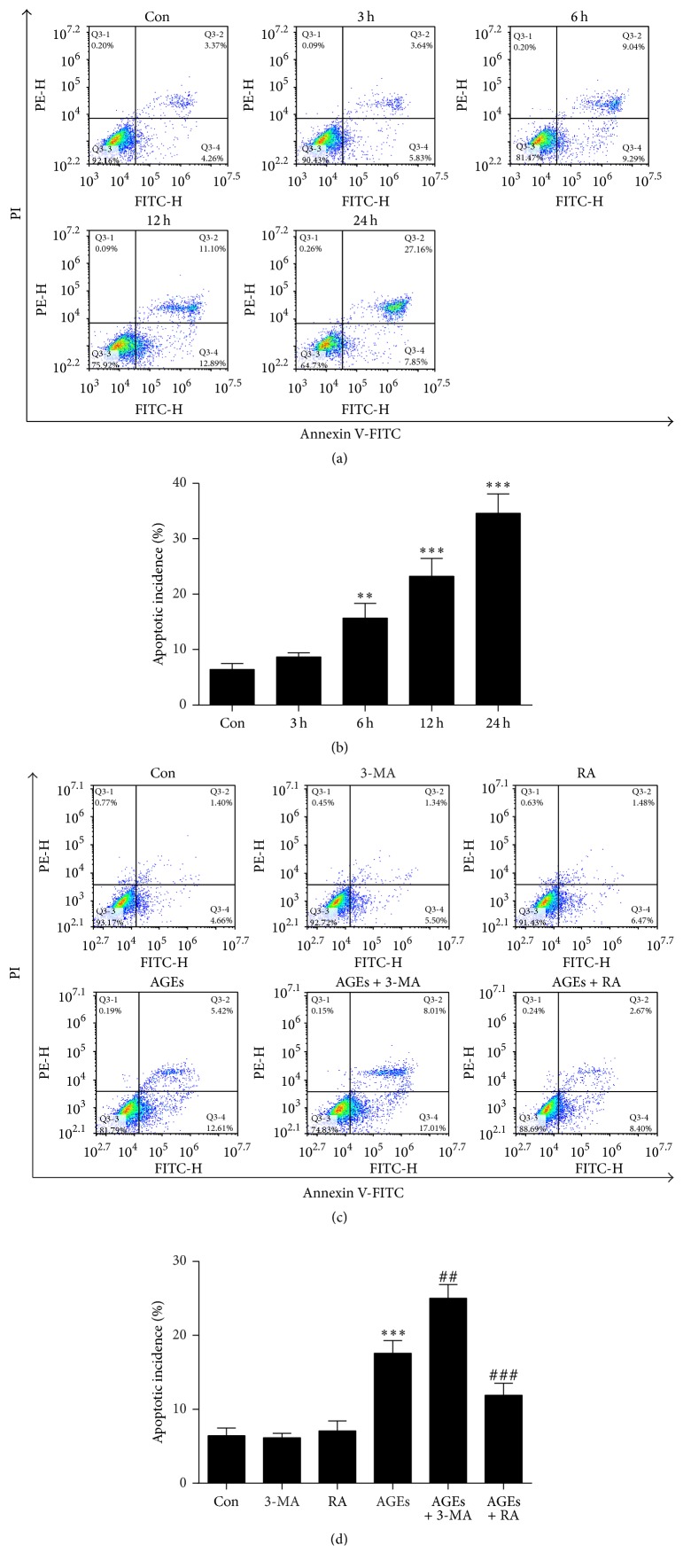
Autophagy against AGE-induced apoptosis in chondrocytes. (a) Apoptosis in chondrocytes was measured by Annexin V/PI double-staining assay after treatment with 100 *μ*g/mL AGEs for 0, 3, 6, 12, and 24 h. (b) Statistical analysis of flow cytometry results for apoptotic percentage of chondrocytes treated with AGEs. (c) Annexin V/PI double-staining assay for the apoptotic proportion of chondrocytes after pretreatment with 3-MA (5 mM) or RA (5 *μ*M) for 1 h, followed by 100 *μ*g/mL AGEs for 6 h. (d) The effect of autophagy on AGE-induced apoptotic rate. All results are the mean ± SD of three independent experiments. ^*∗∗*^*p* < 0.01 and ^*∗∗∗*^*p* < 0.001 versus the control group. ^##^*p* < 0.01 and ^###^*p* < 0.001 versus AGE-treated cells.

To investigate whether AGEs induced apoptotic cell death in chondrocytes, flow cytometric analysis with Annexin V-FITC/PI double-labeling revealed that AGEs significantly increased the number of apoptotic cells (Annexin V+/PI− and Annexin V+/PI+) in a time dependent manner (Figures 3(a) and 3(b)). The chondrocytes were pretreated with 3-MA (5 mM) or RA (5 *μ*M) for 1 h before the treatment of AGEs. Annexin V-FITC/PI double-labeling showed that RA remarkably attenuated AGE-induced apoptosis in chondrocytes, whereas AGE-induced apoptotic cells were increased when chondrocytes were cocultured with 3-MA (Figures 3(c) and 3(d)).

### 3.4. Autophagy Attenuated AGE-Induced MMP-3 and MMP-13 Expression in Chondrocytes

**Figure 4:**
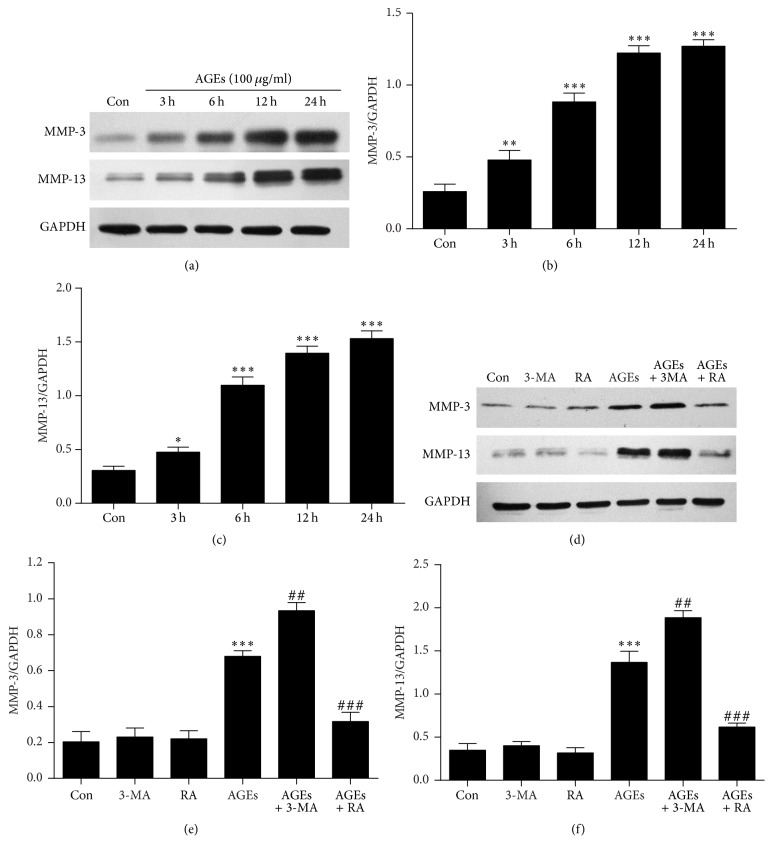
Autophagy plays a protective role in AGE-induced MMP-3 and MMP-13 expression in chondrocytes. (a) MMP-3 and MMP-13 protein levels were measured by Western blot assay after treatment with 100 *μ*g/mL AGEs for 0, 3, 6, 12, and 24 h. (b, c) Protein densitometric quantification of MMP-3 and MMP-13. (d) Chondrocytes were treated with 100 *μ*g/mL AGEs for 6 h with or without pretreatment of 3-MA (5 mM) or RA (5 *μ*M) for 1 h. The protein levels of MMP-3 and MMP-13 are determined by Western blot analysis. (e, f) Protein densitometric quantification of MMP-3 and MMP-13. All results are the mean ± SD of three independent experiments. ^*∗*^*p* < 0.05, ^*∗∗*^*p* < 0.01, and ^*∗∗∗*^*p* < 0.001 versus the control group. ^##^*p* < 0.01 and ^###^*p* < 0.001 versus AGE-treated cells.

Cells were treated with AGEs (100 *μ*g/mL) for various times (0, 3, 6, 12, and 24 h). The expression of MMP-3 and MMP-13 in chondrocytes was elevated after 6 hours of treatment with AGEs (Figures 4(a), 4(b), and 4(c)). To explore the role of autophagy in AGE-induced MMP-3 and MMP-13 expression in chondrocytes, chondrocytes were pretreated with 3-MA (5 mM) or RA (5 *μ*M) 1 h before the addition of AGEs. RA pretreatment prevented the increase of MMP-3 and MMP-13 expression induced by AGEs. In contrast, levels of MMP-3 and MMP-13 were both significantly increased in cells pretreated with 3-MA compared to cells treated with AGEs alone (Figures 4(d), 4(e), and 4(f)).

## 4. Discussion

Osteoarthritis (OA) is a degenerative disease that occurs in hand, hip, and knee joints [2]. OA is mainly characterized by the death of chondrocytes and cartilage degeneration and is becoming more prevalent in the world due to a steady increase in the aging population [19]. AGE originates from endogenous or exogenous sources with aging and is one of the main factors that contributes to the development of OA [5]. The accumulation of AGEs was detected in the cartilage of aged mice, and the elevation of AGEs increased collagen crosslinking, which led to a loss of elasticity and subsequently made cartilage more brittle [20]. However, the underlying mechanisms are not completely understood. It has been reported that AGEs can cause chondrocyte apoptosis and increase MMP expression, which disrupts cartilage homeostasis [6, 8]. Autophagy is a mechanism of cellular homeostasis leading to the degeneration of unnecessary or dysfunctional cellular organelles and closely interacts with apoptosis. Recent research also suggests that autophagy is involved in the pathogenesis of age-related OA [10].

Autophagy can be stimulated by catabolic and nutritional stresses with increased autophagy-related proteins of LC3 and Beclin1 in OA chondrocytes [9]. In the present study, we found that both LC3 and Beclin1 expression in chondrocytes were increased after AGE treatment. In addition, we transfected chondrocytes with mCherry-GFP-LC3B-adenovirus to detect autolysosome. Our results show that AGEs can activate autophagic flux in chondrocytes. Recent studies suggested that autophagy is considered to take part in AGE-associated diseases, such as osteoporotic fracture, cardiomyopathy, and atherosclerosis [14, 15, 21, 22]. However, the relationship between autophagy and those AGE-associated diseases is controversial. Furthermore, the effect of AGEs on autophagy of chondrocytes has not been investigated. It has been proposed that autophagy activation significantly protects from cartilage destruction, proteoglycan loss, and the death of chondrocytes [9]. Based on previous studies, we explore the role of autophagy on AGE-induced apoptosis and MMP expression in chondrocytes.

Apoptosis, known as type-I programmed cell death (PCD), plays an essential role in maintaining homeostasis in multicellular organisms as well as regulating normal embryonic development. Dysregulation of apoptosis leads to a variety of diseases, such as cancer, developmental anomalies, and degenerative diseases involving OA [23]. The chondrocyte is important for maintaining cartilage homeostasis, in part through the production of ECM components [2]. Adult articular cartilage cannot compensate for the loss of chondrocytes occurring in aging during OA [24]. AGEs can induce apoptosis in chondrocytes by mitochondria-mediated caspase-3 activity and activation of MAPK and NF-*κ*B [8, 25]. Consistent with previous studies, our results from Annexin V-FITC/PI double-labeling found AGEs induced apoptosis in rat chondrocytes. The relationship between autophagy and apoptosis is complex. Autophagy can inhibit apoptosis but can sometimes cause autophagic cell death (type II PCD) [26]. Herein, we found that the promotion of autophagy by RA preserved AGE-induced apoptosis in chondrocytes, and conversely, the inhibitor of 3-MA aggravated AGE-induced toxicity. These results confirm that autophagy plays a protective role in the AGE-induced apoptosis of chondrocytes.

The homeostasis of normal cartilage in adults represents a proper balance between anabolism and catabolism of ECM components [5]. AGEs not only generate chondrocyte apoptosis but also increase catabolic activity, causing cartilage matrix breakdown. MMPs are important regulators of the degradation of ECM during OA development. Among the MMPs, MMP-3 and MMP-13 are important for degrading components of ECM, including collagens, proteoglycans, and other extracellular matrix macromolecules in cartilage [27]. The serum level of MMP-3 was found to be elevated with aging in patients with OA [28], and this protease is capable of cleaving proteoglycans [19]. In addition, MMP-3 can indirectly activate pro-MMP-9, a gelatinase [29]. Accordingly, MMP-13 (collagenase-3) is a major type II collagen degrading enzyme [29]. Recent studies elucidated that elevated MMP-13 was noted at the deep zones of arthritic cartilage, and this protease appears to be the major MMP responsible for matrix degradation [30]. AGEs have a critical role in cartilage matrix degradation through stimulating chondrocytes to induce proinflammatory and procatabolic mediators, including MMP-3 and MMP-13 [6, 31]. In the present study, we observed that AGE treatment significantly increased the expression of MMP-3 and MMP-13 in chondrocytes in a dose and time dependent manner. However, this effect could be inhibited by autophagy inducer RA or aggravated by autophagy inhibitor 3-MA. This result suggests that autophagy may alleviate cartilage loss by downregulating AGE-induced MMP-3 and MMP-13 expression.

## 5. Conclusion

In summary, this study demonstrates that autophagy is linked with AGE-related pathological changes of OA, and it plays a protective role in AGE-induced apoptosis and in the upregulation of MMP-3 and MMP-13 in rat chondrocytes. This study provides novel insight into preventive and therapeutic approaches to OA and might potentially lead to a better understanding of the molecular mechanism of age-related OA.

## References

[1] Leong D. J., Sun H. B. (2011). Events in articular chondrocytes with aging. *Current Osteoporosis Reports*.

[2] Li Y., Wei X., Zhou J., Wei L. (2013). The age-related changes in cartilage and osteoarthritis. *BioMed Research International*.

[3] Loeser R. F., Collins J. A., Diekman B. O. (2016). Ageing and the pathogenesis of osteoarthritis. *Nature Reviews Rheumatology*.

[4] Nedić O., Rattan S. I. S., Grune T., Trougakos I. P. (2013). Molecular effects of advanced glycation end products on cell signalling pathways, ageing and pathophysiology. *Free Radical Research*.

[5] Bonet M. L., Granados N., Palou A. (2011). Molecular players at the intersection of obesity and osteoarthritis. *Current Drug Targets*.

[6] Nah S.-S., Choi I.-Y., Yoo B., Kim Y. G., Moon H.-B., Lee C.-K. (2007). Advanced glycation end products increases matrix metalloproteinase-1, -3, and -13, and TNF-*α* in human osteoarthritic chondrocytes. *FEBS Letters*.

[7] Huang C.-Y., Lai K.-Y., Hung L.-F., Wu W.-L., Liu F.-C., Ho L.-J. (2011). Advanced glycation end products cause collagen II reduction by activating Janus kinase/signal transducer and activator of transcription 3 pathway in porcine chondrocytes. *Rheumatology*.

[8] Yang Q., Guo S., Wang S., Qian Y., Tai H., Chen Z. (2015). Advanced glycation end products-induced chondrocyte apoptosis through mitochondrial dysfunction in cultured rabbit chondrocyte. *Fundamental and Clinical Pharmacology*.

[9] Li Y.-S., Zhang F.-J., Zeng C. (2016). Autophagy in osteoarthritis. *Joint Bone Spine*.

[10] Caramés B., Olmer M., Kiosses W. B., Lotz M. K. (2015). The relationship of autophagy defects to cartilage damage during joint aging in a mouse model. *Arthritis and Rheumatology*.

[11] Shi J., Zhang C., Yi Z., Lan C. (2016). Explore the variation of MMP3, JNK, p38 MAPKs, and autophagy at the early stage of osteoarthritis. *IUBMB Life*.

[12] Takayama K., Kawakami Y., Kobayashi M. (2014). Local intra-articular injection of rapamycin delays articular cartilage degeneration in a murine model of osteoarthritis. *Arthritis research & therapy*.

[13] Vasheghani F., Zhang Y., Li Y.-H. (2015). PPAR*γ* deficiency results in severe, accelerated osteoarthritis associated with aberrant mTOR signalling in the articular cartilage. *Annals of the Rheumatic Diseases*.

[14] Hu P., Zhou H., Lu M. (2015). Autophagy plays a protective role in advanced glycation end product-induced apoptosis in cardiomyocytes. *Cellular Physiology and Biochemistry*.

[15] Yang L., Meng H., Yang M. (2016). Autophagy protects osteoblasts from advanced glycation end products-induced apoptosis through intracellular reactive oxygen species. *Journal of Molecular Endocrinology*.

[16] Caramés B., Taniguchi N., Otsuki S., Blanco F. J., Lotz M. (2010). Autophagy is a protective mechanism in normal cartilage, and its aging-related loss is linked with cell death and osteoarthritis. *Arthritis and Rheumatism*.

[17] Chen J.-W., Ni B.-B., Li B., Yang Y.-H., Jiang S.-D., Jiang L.-S. (2014). The responses of autophagy and apoptosis to oxidative stress in nucleus pulposus cells: implications for disc degeneration. *Cellular Physiology and Biochemistry*.

[18] Klionsky D. J., Abdalla F. C., Abeliovich H. (2012). Guidelines for the use and interpretation of assays for monitoring autophagy. *Autophagy*.

[19] Wieland H. A., Michaelis M., Kirschbaum B. J., Rudolphi K. A. (2005). Osteoarthritis—an untreatable disease?. *Nature Reviews Drug Discovery*.

[20] Kim J.-H., Lee G., Won Y. (2015). Matrix cross-linking-mediated mechanotransduction promotes posttraumatic osteoarthritis. *Proceedings of the National Academy of Sciences of the United States of America*.

[21] Hou X., Hu Z., Xu H. (2014). Advanced glycation endproducts trigger autophagy in cadiomyocyte via RAGE/PI3K/AKT/mTOR pathway. *Cardiovascular Diabetology*.

[22] Xie Y., You S.-J., Zhang Y.-L. (2011). Protective role of autophagy in AGE-induced early injury of human vascular endothelial cells. *Molecular Medicine Reports*.

[23] Hwang H. S., Kim H. A. (2015). Chondrocyte apoptosis in the pathogenesis of osteoarthritis. *International Journal of Molecular Sciences*.

[24] Zhang X.-H., Xu X.-X., Xu T. (2015). Ginsenoside Ro suppresses interleukin-1*β*-induced apoptosis and inflammation in rat chondrocytes by inhibiting NF-*κ*B. *Chinese Journal of Natural Medicines*.

[25] Zhang H.-B., Zhang Y., Chen C., Li Y.-Q., Ma C., Wang Z.-J. (2016). Pioglitazone inhibits advanced glycation end product-induced matrix metalloproteinases and apoptosis by suppressing the activation of MAPK and NF-*κ*B. *Apoptosis*.

[26] Li G., Wang G., Ma L. (2016). miR-22 regulates starvation-induced autophagy and apoptosis in cardiomyocytes by targeting p38*α*. *Biochemical and Biophysical Research Communications*.

[27] Zhou K., Hu L., Liao W., Yin D., Rui F. (2016). Coptisine prevented IL-*β*-induced expression of inflammatory mediators in chondrocytes. *Inflammation*.

[28] Manicourt D.-H., Fujimoto N., Obata K., Thonar E. J.-M. A. (1994). Serum levels of collagenase, stromelysin-1, and TIMP-1. Age- and sex-related differences in normal subjects and relationship to the extent of joint involvement and serum levels of antigenic keratan sulfate in patients with osteoarthritis. *Arthritis and Rheumatism*.

[29] Bramono D. S., Richmond J. C., Weitzel P. P., Kaplan D. L., Altman G. H. (2004). Matrix metalloproteinases and their clinical applications in orthopaedics. *Clinical Orthopaedics and Related Research*.

[30] Drinda S., Franke S., Canet C. C. (2002). Identification of the advanced glycation end products N*ε*-carboxymethyllysine in the synovial tissue of patients with rheumatoid arthritis. *Annals of the Rheumatic Diseases*.

[31] Nah S.-S., Choi I.-Y., Lee C. K. (2008). Effects of advanced glycation end products on the expression of COX-2, PGE2 and NO in human osteoarthritic chondrocytes. *Rheumatology*.

